# Cdk1 Targets Srs2 to Complete Synthesis-Dependent Strand Annealing and to Promote Recombinational Repair

**DOI:** 10.1371/journal.pgen.1000858

**Published:** 2010-02-26

**Authors:** Marco Saponaro, Devon Callahan, Xiuzhong Zheng, Lumir Krejci, James E. Haber, Hannah L. Klein, Giordano Liberi

**Affiliations:** 1 Institute of Molecular Oncology Foundation, The Italian Foundation for Cancer Research and Dipartimento di Scienze Biomolecolari e Biotecnologie–University of Milan, Milan, Italy; 2 Department of Biochemistry and Kaplan Cancer Center, New York University School of Medicine, New York, New York, United States of America; 3 Department of Biology and National Center for Biomolecular Research, Masaryk University, Brno, Czech Republic; 4 Department of Biology and Rosenstiel Medical Center, Brandeis University, Waltham, Massachusetts, United States of America; The University of North Carolina at Chapel Hill, United States of America

## Abstract

Cdk1 kinase phosphorylates budding yeast Srs2, a member of UvrD protein family, displays both DNA translocation and DNA unwinding activities *in vitro*. Srs2 prevents homologous recombination by dismantling Rad51 filaments and is also required for double-strand break (DSB) repair. Here we examine the biological significance of Cdk1-dependent phosphorylation of Srs2, using mutants that constitutively express the phosphorylated or unphosphorylated protein isoforms. We found that Cdk1 targets Srs2 to repair DSB and, in particular, to complete synthesis-dependent strand annealing, likely controlling the disassembly of a D-loop intermediate. Cdk1-dependent phosphorylation controls turnover of Srs2 at the invading strand; and, in absence of this modification, the turnover of Rad51 is not affected. Further analysis of the recombination phenotypes of the *srs2* phospho-mutants showed that Srs2 phosphorylation is not required for the removal of toxic Rad51 nucleofilaments, although it is essential for cell survival, when DNA breaks are channeled into homologous recombinational repair. Cdk1-targeted Srs2 displays a PCNA–independent role and appears to have an attenuated ability to inhibit recombination. Finally, the recombination defects of unphosphorylatable Srs2 are primarily due to unscheduled accumulation of the Srs2 protein in a sumoylated form. Thus, the Srs2 anti-recombination function in removing toxic Rad51 filaments is genetically separable from its role in promoting recombinational repair, which depends exclusively on Cdk1-dependent phosphorylation. We suggest that Cdk1 kinase counteracts unscheduled sumoylation of Srs2 and targets Srs2 to dismantle specific DNA structures, such as the D-loops, in a helicase-dependent manner during homologous recombinational repair.

## Author Summary

Broken DNA molecules can be repaired by copying a homologous DNA sequence located elsewhere in the genome. This process, called homologous recombination, needs to be carefully regulated, because unwanted DNA exchanges can lead to genome rearrangements and cell death. Cdk1 kinase is required for cell cycle progression and phosphorylates DNA repair factors, such as Srs2, a protein that can both translocate on single-stranded DNA and open the two strands of DNA double helix. DNA translocation activity of Srs2 is crucial to prevent unwanted recombination, while DNA unwinding activity might be important to promote recombination. In this study, we used two *srs2* mutants that constitutively express the unphosphorylated or Cdk1-dependent phosphorylated Srs2 protein isoforms. We found that Srs2 performs genetically distinct functions in preventing or promoting homologous recombination. Cdk1 targets Srs2 to promote accurate repair of double-stranded DNA breaks, but is not essential for the removal of toxic recombination intermediates assembled at single-stranded DNA breaks. Further, Cdk1 counteracts sumoylation of Srs2, which is responsible for recombination defects due to the lack of Srs2 phosphorylation. In summary, Cdk1-dependent Srs2 phosphorylation prevents its unscheduled sumoylation and targets the helicase to promote accurate homologous recombinational repair.

## Introduction

Homologous recombination (HR) is a fundamental DNA repair pathway and its deregulation is responsible for a variety of genomic rearrangements, including chromosome loss, DNA translocations and inversions, which are typical of the genetic alterations seen in tumor cells (reviewed in [1]). The mechanisms and proteins involved in HR have been well conserved throughout evolution and much of our knowledge on HR comes from studies conducted in the yeast *Saccharomyces cerevisiae* (reviewed in [2]–[5]). HR targets multiple DNA lesions, including single-stranded DNA (sDNA) breaks and DSBs, promoting their repair using a region of homology as a template. Diverse pathways can seal sDNA breaks, but the role of HR in DSB repair is essential. Different HR sub-pathways compete for DSB repair and some are less accurate than others [6],[7]. The position of DNA sequences involved in recombination and the extent of their homology influence the kinetics of DSB repair. Irreparable DNA breaks [8], or even those repaired slowly [9], appear to be sequestered to the nuclear periphery, through a mechanism resembling that used to tether telomeres at the nuclear membrane [10]. When a region of homology is found on both sides of a DSB, the preferred pathway of repair is gene conversion (GC). Among the initial steps in GC is the formation of Rad51 presynaptic nucleofilaments assisted by accessory factors. While Rad51 nucleation can occur directly at sDNA breaks, the ends of DSBs must be first processed to produce sDNA tails in order to recruit Rad51. Multiple factors with nuclease and/or helicase activities, including the Mre11/Rad50/Xrs2 complex, Sae2, Exo1, Dna2 and Sgs1 cooperate in 5′ to 3′ DSB resection (reviewed in [11]). Assembled Rad51 nucleofilaments invade and displace a duplex donor homologue DNA template leading to the formation of a D-loop structure. The D-loop is the site of DNA synthesis, which is promoted by extension of the 3′ invading strand. According to the canonical DSB repair model [12], the capture of the second end of the DSB generates a double Holliday junction (dHJ) whose resolution, by cutting or branch migration, influences the formation of crossover products associated with GC, that is, the extent of DNA exchanges associated with DSB repair. If the second DSB end is not captured, it can anneal to the invading strand evicted from the D-loop soon after DNA synthesis. In this process, called synthesis-dependent strand annealing (SDSA), GC is limited to DNA synthesized from donor strand and crossovers are prevented [4]. Another HR pathway, known as single strand annealing (SSA), is used when DSB repair occurs between direct repeats [4]. In this case resected homologous sequences anneal without DNA synthesis and DSB repair is associated with deletion of the sequence between the repeats. Notably, during SSA, a D-loop is not formed and Rad51 is not required. The formation of presynaptic Rad51 nucleofilaments is fundamental for HR commitment during GC. However, Rad51 could nucleate improperly on DNA or even be engaged into damaged filaments when other recombination factors are inactivated: in both cases HR is not proficient, rather it becomes toxic for other DNA transactions.

Many *in vivo* studies suggest that Srs2, a member of UvrD family of DNA helicases conserved from bacteria to human, is involved in the removal of toxic Rad51 filaments from sDNA [13]–[17]. Further, the Srs2 protein disrupts presynaptic Rad51 filaments through its DNA translocase activity *in vitro* [18],[19]. This Srs2 anti-recombination activity requires a physical interaction with sumoylated PCNA, as it was evidenced in the absence of the post-replication repair (PRR) pathway, a context in which Srs2 prevents deadly the recombinational repair [20],[21]. Srs2 also exhibits 3′ to 5′ DNA helicase activity on duplex DNA [22]. Recent *in vitro* studies in yeast and plants suggest that Srs2 unwinds DNA structures mimicking a D-loop [23],[24]. Genetic evidence, indeed, suggests that Srs2 favors the SDSA pathway, since the loss of Srs2 results in an increase in crossover products [25]–[27]. Moreover, Srs2 is essential for DSB repair through either SSA or ectopic GC [25],[28],[29]; in SSA repair, Srs2 is required to mediate recovery from checkpoint-mediated arrest [29].

Since Srs2 affects HR in several ways, Srs2 functions in recombination are probably regulated. Previous studies demonstrated that Srs2 is a target of the cell cycle-dependent kinase (Cdk1) *in vivo* [30] and *in vitro* [31]. Cdk1 has been implicated in the DNA damage response and in DSB repair [32]; by monitoring repair of one HO-induced break, it was shown that Cdk1 is required both at the level of resection and at a step after Rad51-dependent strand invasion [33],[34]. It is known that Cdk1 triggers the resection of DSB ends by phosphorylating Sae2 [35], but other direct targets in DSB repair are unknown.

We found that *srs2* mutants that are unable to undergo Cdk1-dependent phosphorylation can still remove toxic Rad51 nucleofilaments, but these *srs2* mutants fail to promote homologous recombinational repair. Analysis on repair of a single HO-induced break through ectopic GC shows that the proper turnover of Srs2, at D-loop intermediates, is dependent on its modification by phosphorylation and this phosphorylation is essential for completion of the SDSA reaction that results in non-crossover products. Moreover, the phosphorylation-dependent role of Srs2 does not require an interaction with PCNA and does not affect the turnover of Rad51 at invading filaments. In the absence of Srs2 phosphorylation, the protein is sumoylated and this is the main cause of the recombinational repair defects seen in the nonphosphorylatable *srs2* mutant. Thus, coordination of the sumoyaltion and phosphorylation modifications on Srs2 is essential during homologous recombinational repair.

## Results

### The C-terminus tail of Srs2 contains consensus sites for Cdk1-dependent phosphorylation and sumoylation

**Figure 1:**
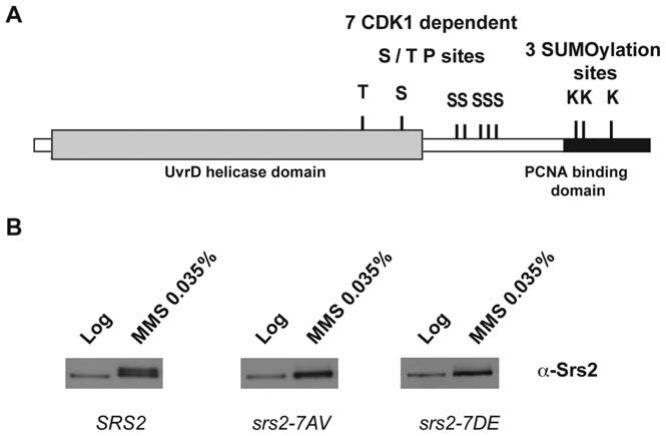
Srs2 contains consensus sites for multiple post-translational modifications. (A) Schematic view of Srs2 with the conserved UvrD helicase domain (light grey box) and the PCNA binding domain that is composed of the last 138 amino acids of the protein (black box). Srs2 contains seven CDK1-dependent consensus sites and, within the PCNA binding domain, three sumoylation consensus sites. (B) Protein extracts were prepared from cultures of *SRS2* and *srs2* phospho-mutants strains grown in log-phase with or without MMS and analyzed using anti-Srs2 antibodies to assess protein levels and phosphorylation status.

*Saccharomyces cerevisiae* Srs2 contains characteristic amino acid motifs important for ATP-binding and DNA-binding that are highly conserved among members of UvrD family [36]. All these motifs are located in the N-terminal domain of the Srs2 protein (grey box in Figure 1A) and are sufficient for the helicase activity [22], but not for translocase-dependent removal of Rad51 nucleofilaments, as tested *in vitro* [37],[38]. The C-terminal tail of Srs2 protein plays an important regulatory function, since it mediates protein-protein interactions, including interaction with Rad51 and PCNA [15], [21], [37]–[40]. Moreover, a cluster of five consensus sites for Cdk1 kinase is present in the C-terminal region of Srs2, while two additional sites are located in the helicase domain (Figure 1A; [39]). The last 138 amino acids (aa) of the Srs2 C-terminal tail are required for the interaction with PCNA [21] and also contain three consensus sites for sumoylation (Figure 1A).

We previously showed that changing the seven serine/threonine Cdk1 consensus sites to the unphosphorylatable residues alanine/valine abolished DNA damage-induced phosphorylation of Srs2, which can be monitored as an electrophoretic mobility shift on SDS-PAGE (Figure 1B; [39]). We then produced a new *srs2* allele in which the same serine/threonine residues were changed to the negatively charged aspartic acid/glutamic acid residues, with the aim of producing a mutated version of Srs2 that mimics the constitutively phosphorylated protein isoform. As shown in Figure 1B, the levels of wt Srs2 and the two mutated Srs2 isoforms are similar, both in normal conditions and in response to DNA damage by methyl methanesulfonate (MMS)-treatment (data not shown). Henceforth, we will refer to the unphosphorylated and phosphorylated *srs2* mutants, respectively, as *srs2-7AV* and *srs2-7DE*.

### Srs2 phosphorylation promotes recombinational repair, but is not essential for the reversal of toxic Rad51 nucleofilaments accumulating at sDNA gaps

**Figure 2:**
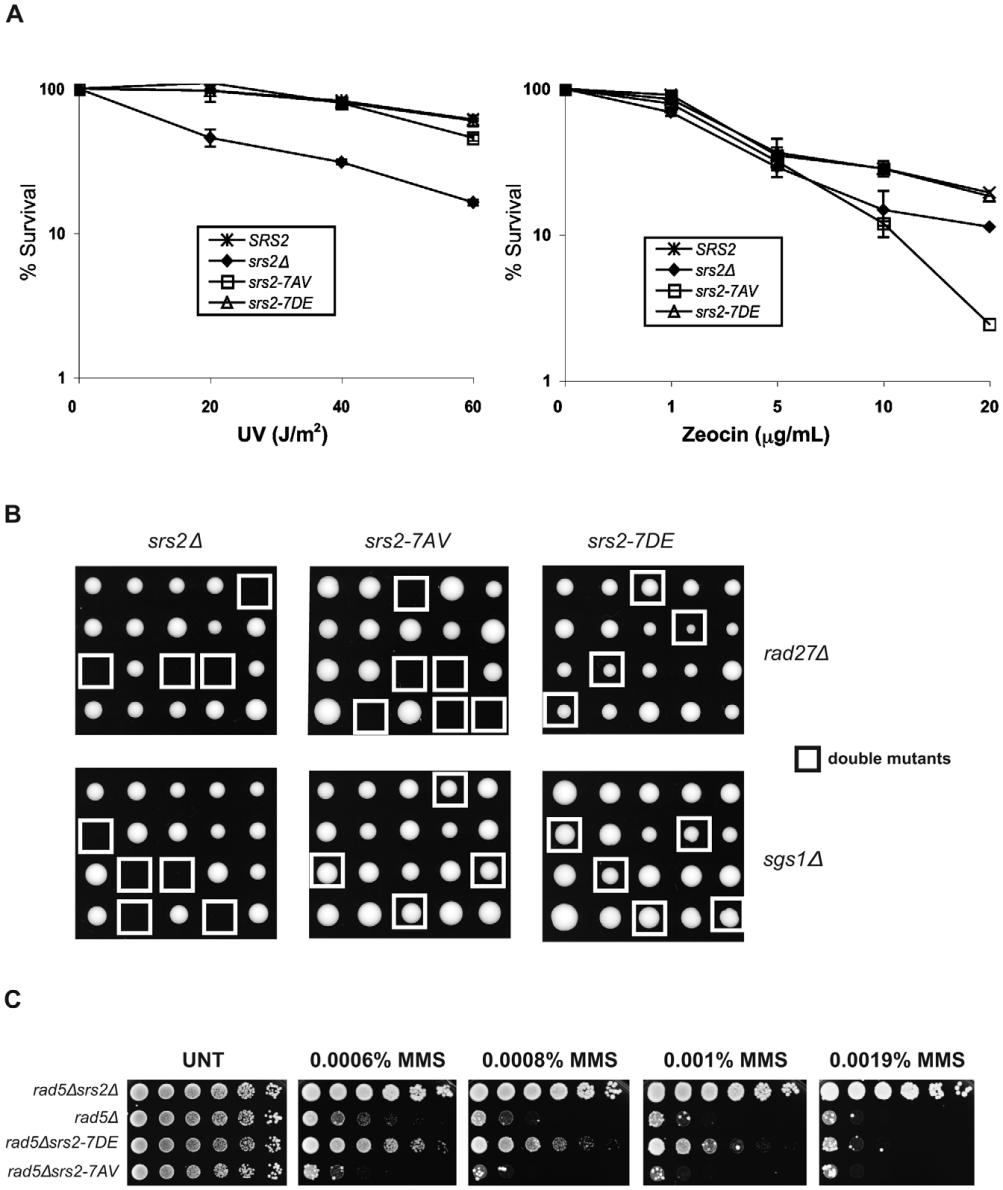
Phosphorylation of Srs2 is required for cell survival when DNA break repair is channeled into HR. (A) Survival of *SRS2*, *srs2Δ*, *srs2-7AV*, and *srs2-7DE* strains was determined after exposure to different doses of UV-light and the DSB-inducer zeocin. (B) Tetrads obtained from sporulation of diploids heterozygous for the indicated mutations. Double mutant spores are indicated by the white squares. (C) The indicated strains were grown at an equal cell concentration, sequentially diluted 1:6 and spotted onto plates containing MMS at the indicated concentrations. Cell growth was evaluated after incubation at 28°C for 3 days.

To investigate whether Cdk1-dependent phosphorylation of Srs2 is important for its roles in HR, we first evaluated cell survival of the two *srs2* phospho-mutants following UV-light and zeocin treatments. Wild type (*SRS2*) and *srs2Δ* strains were used as controls. Previous studies have shown that the UV-sensitivity of *srs2Δ* strains is suppressed by mutations in *RAD51*, indicating that cell lethality is due to accumulation of toxic Rad51 nucleofilaments at gaps whose repair can occur in the absence of HR [16]. We found that *srs2Δ* and *rad51Δ* mutants are also sensitive to zeocin, a radiomimetic chemical that induces DSBs (Figure 2A and data not shown). Thus, zeocin-treatment induces DNA lesions whose repair is strictly HR-dependent and prevented in the absence of Srs2. As shown in Figure 2A, we found that both *srs2-7AV* and *srs2-7DE* mutants, as *SRS2* strains, survive UV-light doses that kill *srs2Δ* mutants. Conversely, the *srs2-7AV* mutant, but not the *srs2-7DE* mutant, is sensitive to zeocin and, indeed, is even more sensitive than the *srs2Δ* strain. Previous reports showed that *srs2Δ* mutations are synthetically lethal with either *sgs1*Δ or *rad27*Δ mutations [13],[14],[41]. While the synthetic lethality of *srs2*Δ *sgs1*Δ double mutants is suppressed by *rad51*Δ [13], single *rad27*Δ mutants are themselves lethal in combination with *rad51*Δ [42]. Thus, the types of spontaneous DNA damage accumulating in *sgs1*Δ and *rad27*Δ mutants mirror those induced by UV and zeocin treatments: only in *rad27*Δ mutants and under zeocin treatment, HR is essential for DNA repair. We crossed the *srs2-7AV* and *srs2-7DE* phospho-mutants and *srs2Δ* as control with *sgs1Δ* or *rad27Δ* mutants. Heterozygous diploid mutants were sporulated and tetrad analysis was performed. As shown in Figure 2B, neither *srs2*Δ *sgs1*Δ nor *srs2*Δ *rad27*Δ double mutants form viable spores; the *srs2-7AV* mutation, but not the *srs2-7DE* mutation, is synthetically lethal with the *rad27*Δ mutation, while both *srs2-7AV sgs1*Δ mutants and *srs2-7DE sgs1*Δ mutant spores form colonies.

Hence, the phenotypes of *srs2-7AV* mutants suggest that Srs2 phosphorylation is dispensable for the reversal of toxic Rad51-dependent recombination intermediates induced at sDNA by UV or by the absence of Sgs1, but phosphorylation is required to promote recombinational repair in zeocin and in the absence of Rad27.

Previous data suggested that the Srs2 protein sensitizes postreplication repair (PRR) mutants, because it prevents HR [43],[44]. Accordingly, as show in Figure 2C, the sensitivity of *rad5*Δ mutants to MMS is alleviated by deleting *SRS2*. *srs2* mutants encoding a protein that displays attenuated translocase activity also suppress the DNA damage sensitivity of PRR mutants, although they are not sensitive to DNA damaging agents by themselves [40]. Hence, we analyzed the *srs2* phospho-mutants in a PRR mutant context, in which the importance of having an intact DNA translocation activity should be revealed. We constructed *srs2* phospho-mutants in *rad5*Δ or *rad18*Δ backgrounds and then tested viability on medium containing MMS. We found that *srs2-7AV* mutation hypersensitizes *rad5*Δ and *rad18*Δ mutants to DNA damage, but, conversely, the *srs2-7DE* mutation partially suppresses the lethality of *rad5*Δ or *rad18*Δ mutation (Figure 2C and data not shown). Notably, *srs2-7AV* and *srs2-7DE* mutants are not sensitive to MMS, even at a higher MMS dose than those employed in Figure 2C (data not shown).

Thus, we conclude that, even in a PRR context, unphosphorylatable Srs2 can remove Rad51 at DNA gaps. On the other hand, the phosphorylated Srs2 protein isoform appears to be less proficient in the anti-recombinational role.

### Srs2 phosphorylation is required for Rad51-dependent DSB repair

The observation that *srs2-7AV* mutants are sensitive to treatment with zeocin suggests that phosphorylation of Srs2 is important in DSB repair. To directly examine this, we tested the behavior of *srs2* phospho-mutants in response to a single DSB created by a galactose-inducible HO endonuclease. Previous studies have shown that *srs2Δ* mutants can not survive a single HO-induced DSB when repair of this break occurs either by ectopic GC or by SSA [25],[28],[29]. While the GC pathway strictly depends on *RAD51*, SSA can occur in the absence of Rad51. There are important differences in the requirement for Srs2 in the two pathways: Srs2 is not required to complete DSB repair during SSA, but it is required for recovery from the DNA damage-induced cell cycle arrest [29]. *RAD51* deletion rescues the checkpoint recovery defect in *srs2Δ* mutants [29]; thus, one hypothesis is that Rad51 accumulates on DNA contributing to the lethal checkpoint-induced arrest, since it can not be removed in absence of Srs2 [29],[45]. Conversely, during ectopic GC, *srs2Δ* mutants are unable to complete DSB repair, with a specific reduction in non-crossover products formation [25]. Since the region of DNA homology involved is limited in ectopic DSB repair, the formation of crossovers might be prevented because the formation of the dHJ intermediate is reduced [46]. Thus, the failure to carry out SDSA results in loss of non-crossover products and there is a marked reduction in DSB repair efficiency [25].

**Figure 3:**
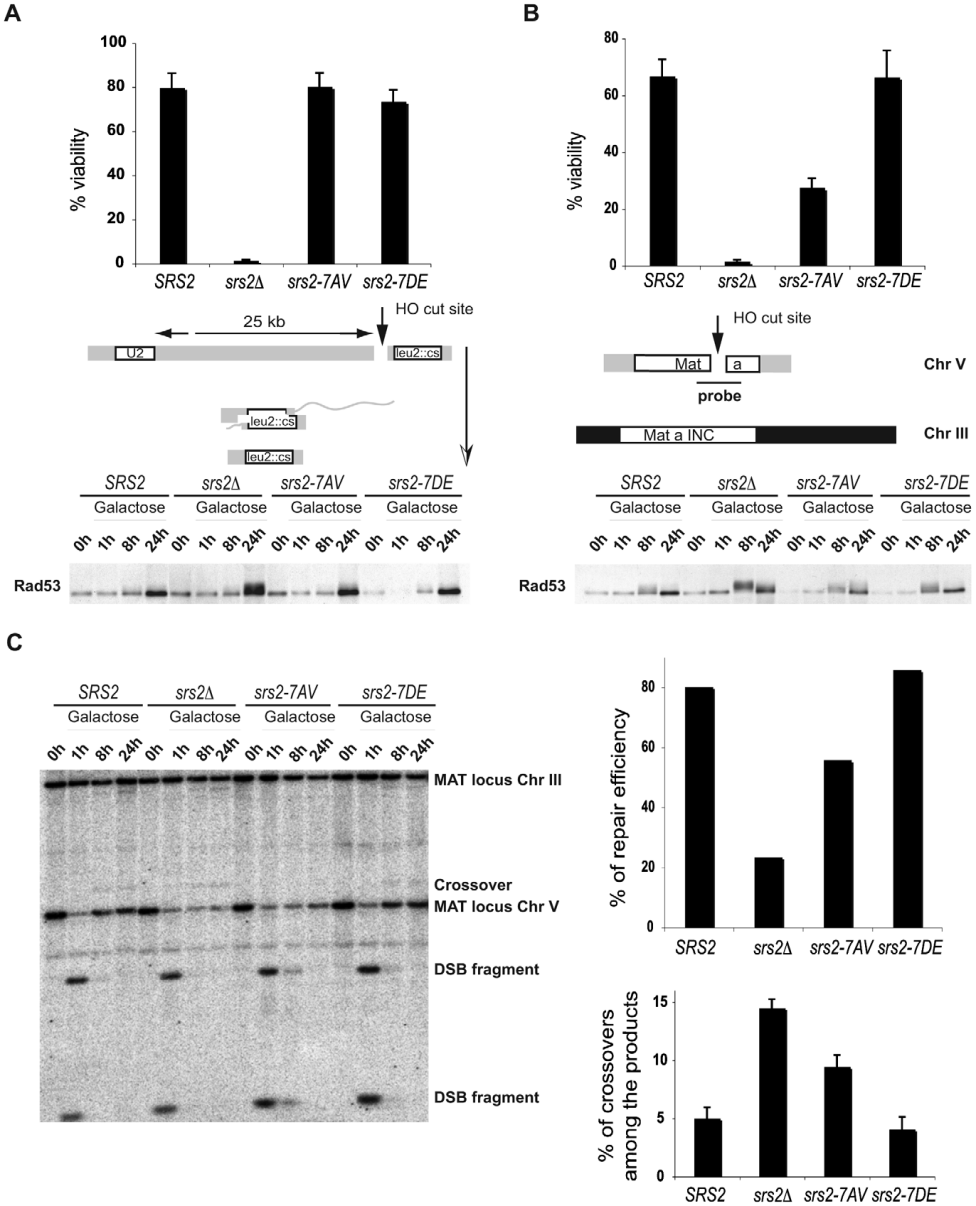
Phosphorylation of Srs2 is required for Rad51-dependent DSB repair via the SDSA pathway. An HO-mediated DSB was induced by addition of galactose to cultures of *SRS2* and *srs2* mutants. Cell viability and Rad53 phosphorylation was analyzed during DSB repair by SSA (A) or GC (B). (C) Southern Blot analysis was performed on EcoRI digested DNA extracted from *SRS2* and *srs2* strains at the indicated time points following galactose induction of the DSB which is repaired by GC. DSB repair efficiency and the percentage of crossover products were calculated at 24 hours after DSB induction.

To analyze the requirement of Srs2 phosphorylation in the DSB repair response, we assayed cell survival of *srs2* phospho-mutants in a SSA system in which DSB repair occurs between repeated sequences, one of which is located 25kb from the DSB and results in a chromosomal deletion [29] or in an ectopic GC system in which DSB repair occurs between chromosomes V and III [25]. In agreement with previous findings, the rate of cell survival of *srs2Δ* mutants is 2% in both the SSA and GC systems (Figure 3A and 3B). This high cell lethality in *srs2Δ* mutants correlates with inability to dephosphorylate the checkpoint kinase Rad53, which is activated in response to DSB induction (Figure 3A and 3B). Cell survival of *srs2-7AV* mutants is 25% in the GC system where they also fail to fully dephosphorylate Rad53 24 hours after DSB induction (Figure 3A and 3B). Survival of the *srs2-7AV* mutant is normal in the SSA system and survival of the *srs2-7DE* mutant is normal in both systems. Thus, Srs2 phosphorylation is necessary for cell survival when DSB repair proceeds through the Rad51-dependent GC pathway, but is dispensable in the SSA pathway, which does not require Rad51. As mentioned above, although SSA is Rad51-independent pathway, in absence of Srs2, Rad51 might improperly accumulate on DNA and interfere with checkpoint recovery [29],[45]. Since *srs2-7AV* survive DSB repair via SSA, this further strengthens the conclusion that Srs2 phosphorylation is not required for reversal of toxic Rad51-dependent intermediates.

We used Southern blotting with a probe that recognizes the *MAT* locus, to physically observe DSB repair in the GC system (Figure 3B). As mentioned above, in this system a DSB is induced at a *MAT* locus inserted into Chromosome V and is repaired using a unique uncleavable *MAT*-inc cassette on chromosome III (Figure 3B). Notably, crossovers that are associated with the GC event can be evaluated by restriction analysis, since crossovers give rise to chromosomal bands that differ in size from the parental chromosomes and the non-crossover GC products (Figure 3B). As shown in Figure 3C, DNA of *SRS2* and *srs2* mutants were analyzed by Southern blotting. DSB repair efficiency is about 30% in *srs2Δ* strain, in agreement with previous findings [25] and in *srs2-7AV* it is reduced to 70% compared to *SRS2* or *srs2-7DE* (Figure 3C). Moreover, the percentage of crossovers associated with GC increases three-fold in *srs2Δ* and two-fold in *srs2-7AV* compared to *SRS2* or *srs2-7DE* (Figure 3C). Similar to the *srs2Δ* mutants, the increase in crossovers is associated with a reduction in non-crossover repair efficiency in the *srs2-7AV* mutant (Figure 3C); thus, DSB repair defects in the absence of Srs2 phosphorylation likely indicate a specific failure to carry out repair via the SDSA pathway that results in non-crossover products.

### Srs2 phosphorylation affects turnover of Srs2, but not turnover of Rad51, during strand invasion in DSB repair

**Figure 4:**
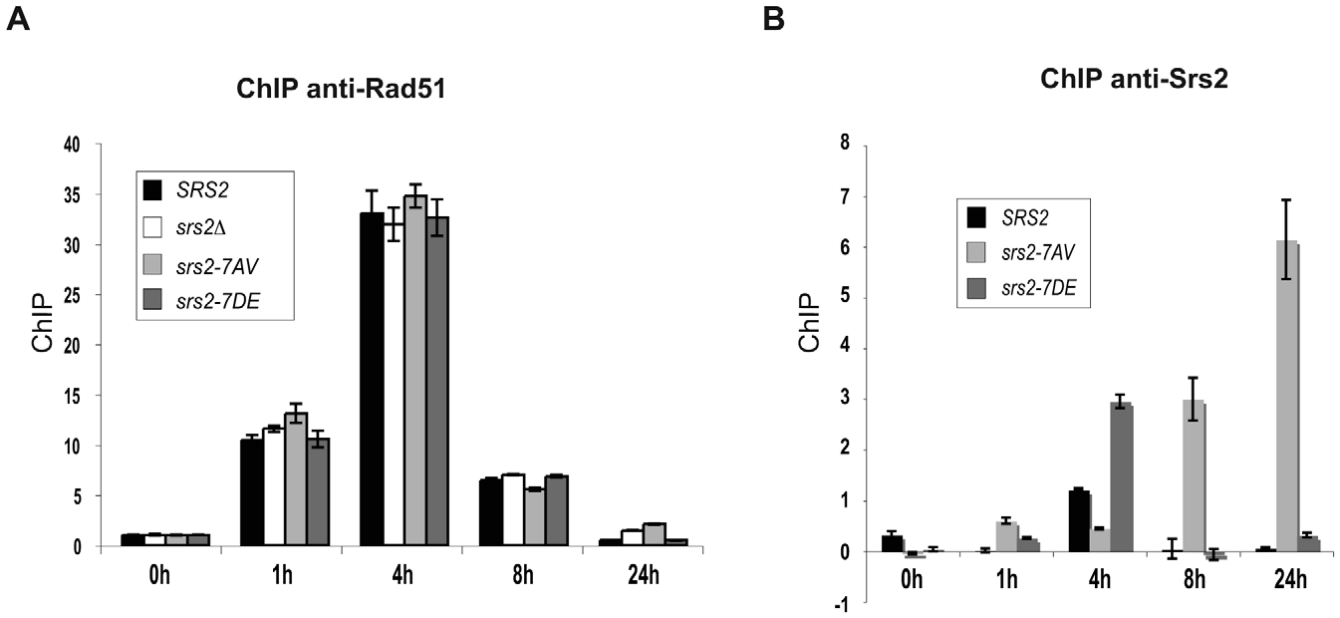
Srs2 phosphorylation controls turnover of Srs2 but not Rad51 at the invading strand during DSB repair. Proteins extracts were prepared from the indicated strains, fixed in formaldehyde and analyzed by ChIP using anti-Rad51 (A) or anti-Srs2 antibodies (B). After DNA cross-linking reversion, real-time PCRs were performed to quantitatively analyze the ChIP results. The DNA region amplified by PCR is located on donor sequence on Chromosome III.

Our analysis indicates that Srs2 phosphorylation is required for Rad51-dependent DSB repair. Although we found that Srs2 phosphorylation is not essential for the removal of toxic Rad51 nucleofilaments at DNA gaps or during DSB repair by SSA, it might be specifically required to remove Rad51-dependent recombination intermediates initiated at D-loop intermediate. To investigate this possibility, we analyzed Rad51 binding to DSBs by ChIP and Q-PCR in *SRS2* and *srs2* phospho-mutants. We used DNA primers that amplified the region of homology located on donor chromosome III. Using this strategy, proteins localizing either at broken or recipient chromosomes will be immunoprecipitated at the DSB when the invading strand is in duplex DNA, which most likely represents the D-loop. As shown in Figure 4A, Rad51 protein is undetectable at the donor *MAT* locus before HO induction, while it is loaded at the DSB with similar kinetics in *SRS2* and all *srs2* mutated strains. Thus, we conclude that Rad51-mediated strand invasion occurs with similar kinetics in *SRS2* and *srs2* mutants. We also conclude that Rad51 is removed from the DSB with similar kinetics in all contexts and strains analyzed. Thus, DSB repair defects in *srs2Δ* or *srs2-7AV* mutants are unrelated to an abnormal persistence of Rad51 after strand invasion.

Previous findings have indicated that Srs2 is loaded at DSBs [47]. We asked whether Srs2 phosphorylation could influence its ability to be recruited to DSBs in our GC system. Using the same ChIP strategy employed above, we found that Srs2 is sited at the invading strand with a three-fold enrichment (Figure 4B). The Srs2 and Srs2-7DE proteins are loaded and dislodged from DNA with kinetics resembling that of Rad51, but the Srs2-7AV protein accumulates only at later times and abnormally persists on DNA for at least 24 hours after DSB induction; notably, Rad51 has been displaced from DNA, when Srs2-7AV protein accumulates (Figure 4).

In summary, the data in Figure 4 suggest that Srs2 is loaded at the D-loop during GC and its proper recruitment is governed by Cdk1-dependent phosphorylation. However, the DSB repair defects in *srs2-7AV* or *srs2Δ* mutants appear not be related to inefficient metabolism of Rad51 nucleofilaments at donor DNA sequences.

### Sumoylation of Srs2 is responsible for the recombination defects in *srs2-7AV* phosphomutants

**Figure 5:**
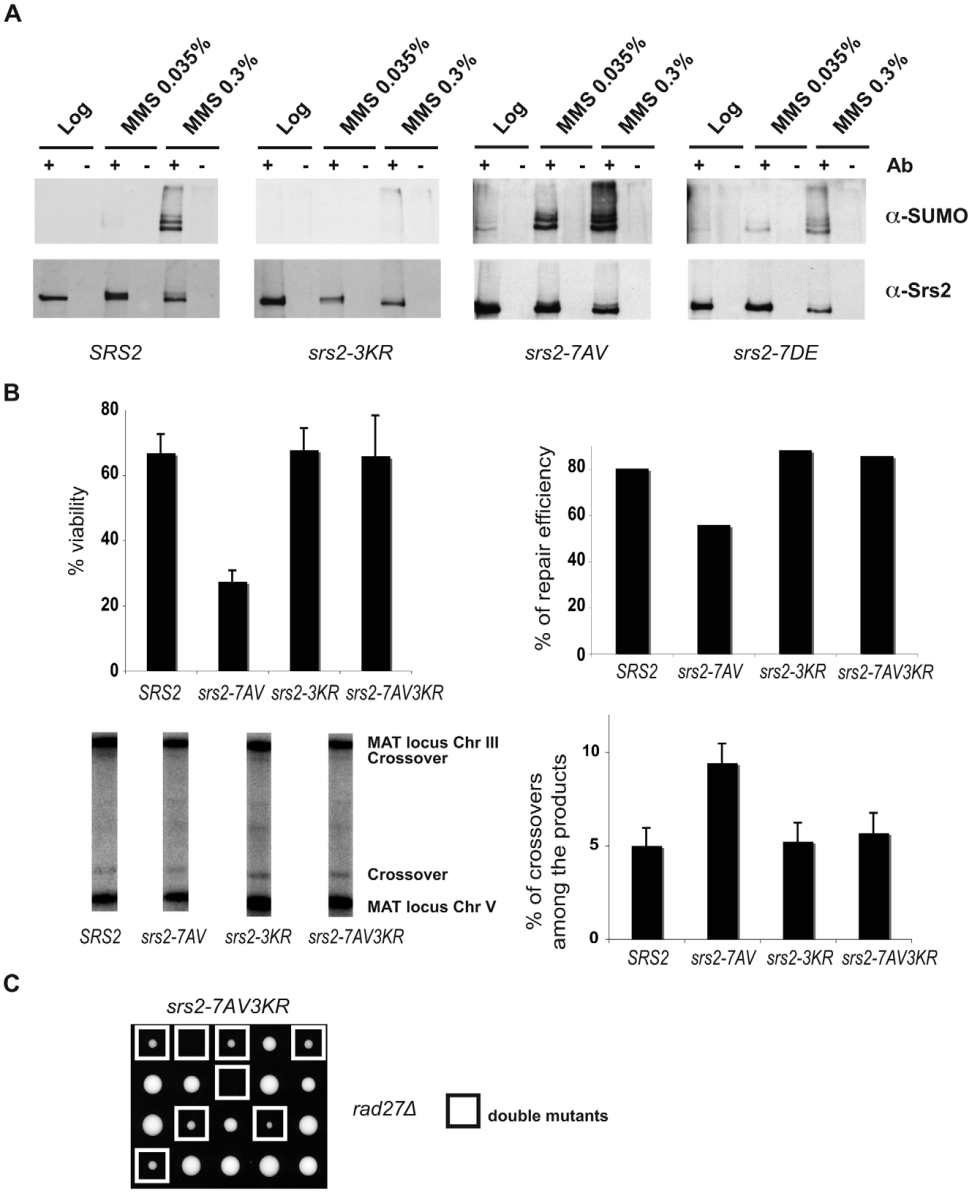
Srs2 sumoylation causes recombination defects in the unphosphorylatable *srs2-7AV* mutant. (A) Srs2 was immunoprecipitated from protein extracts prepared from the indicated yeast strains under DNA damaging conditions. Blots were probed first with anti-SUMO antibodies, then stripped and reprobed with anti-Srs2 antibodies. (B) Cell survival, DSB repair efficiency and rate of crossover formation were determined for the indicated strains as in Figure 3C. (C) Tetrads obtained from sporulation of diploids heterozygous for the *rad27*Δ and *srs2-7AV3KR* mutations. Double mutant spores are indicated by the white squares.

In the course of our studies on Srs2 phosphorylation, we noticed that in response to massive DNA damage, such as treatment with 0.3% MMS, Srs2 accumulates as additional modified isoforms, which can be visualized as a ladder on SDS-PAGE analysis (Figure 5A). These Srs2 protein isoforms are recognized by SUMO-specific antibodies (Figure 5A). Preliminary characterization of Srs2 sumoylation indicates that none of the well-characterized SUMO ligases, including Siz1 and Siz2, are involved in this modification (Figure S1A). Three putative sumoylation sites have been mapped to the C-terminus tail of Srs2 (Figure 1A). Our data indicated that DNA damage-induced sumoylation of Srs2 was abolished in *srs2-3KR* mutants, in which the three lysine residues in the motifs identified as modified by SUMO were mutated to arginine (Figure 5A). Notably, the Srs2-3KR protein can be fully phosphorylated (Figure S1B). Intriguingly, while sumoylation of native Srs2 is induced at 0.3% MMS, the unphosphorylatable Srs2-7AV protein can be detected as SUMO-modified isoforms at ten-fold lower MMS doses (Figure 5A). Thus, while sumoylation and phosphorylation can occur independently, Srs2 accumulates in a sumoylated form in the absence of phosphorylation.

The biological relevance of Srs2 sumoylation is still obscure, as extensive studies of the phenotypes of the *srs2-3KR* mutant were inconclusive (D. Callahan and H. Klein, unpublished results). However, the finding that unphosphorylatable Srs2 is hyper-sumoylated prompted us to ask if the *srs2-7AV* mutant defects in recombinational repair might be related to Srs2 sumoylation. To test this, we mutagenized the sumoylation consensus sites in the *srs2-7AV* mutant to create the *srs2-7AV3KR* allele, which is simultaneously impaired for phosphorylation and sumoylation. We then tested the behavior of the *srs2-7AV3KR* mutant in the DSB repair GC system in which *srs2-7AV* mutant was highly sensitive (see Figure 3). We found that *srs2-7AV3KR* mutant survived DNA damage (Figure 5B); DSB repair is accomplished efficiently and a normal level of crossovers is seen in *srs2-7AV3KR* (Figure 5B). In addition, the *srs2-7AV3KR* mutant correctly reversed the checkpoint response after DSB induction and repair, as seen by Rad53 kinase dephosphorylation (data not shown). Furthermore, the *srs2-3KR* mutant, which is only impaired in sumoylation, can accomplish DSB repair (Figure 5B). To see if ablation of Srs2 sumoylation rescues the phosphorylation defects in recombinational repair in other contexts, we crossed the *srs2-7AV3KR* mutant with the *rad27*Δ mutant to generate *rad27*Δ *srs2-7AV3KR* double mutants. While the *rad27*Δ *srs2-7AV* double mutants never form viable spores (see Figure 2A), we found that *rad27*Δ *srs2-7AV3KR* double mutants developed into visible colonies (17/25 of total cases analyzed), although the double mutant grew very slowly (Figure 5C). This partial suppression highlights the importance of Srs2 protein modifications when it is likely that more than one lesion is formed.

Taken together, the data in Figure 5 indicate that Srs2 is sumoylated *in vivo*. Sumoylation of Srs2 is not required for DSB repair, but the recombinational repair defects in unphosphorylatable *srs2-7AV* mutants are largely related to the unscheduled sumoylation of the protein.

### Cdk1-targeted role of Srs2 is exerted independently of its interaction with PCNA

**Figure 6:**
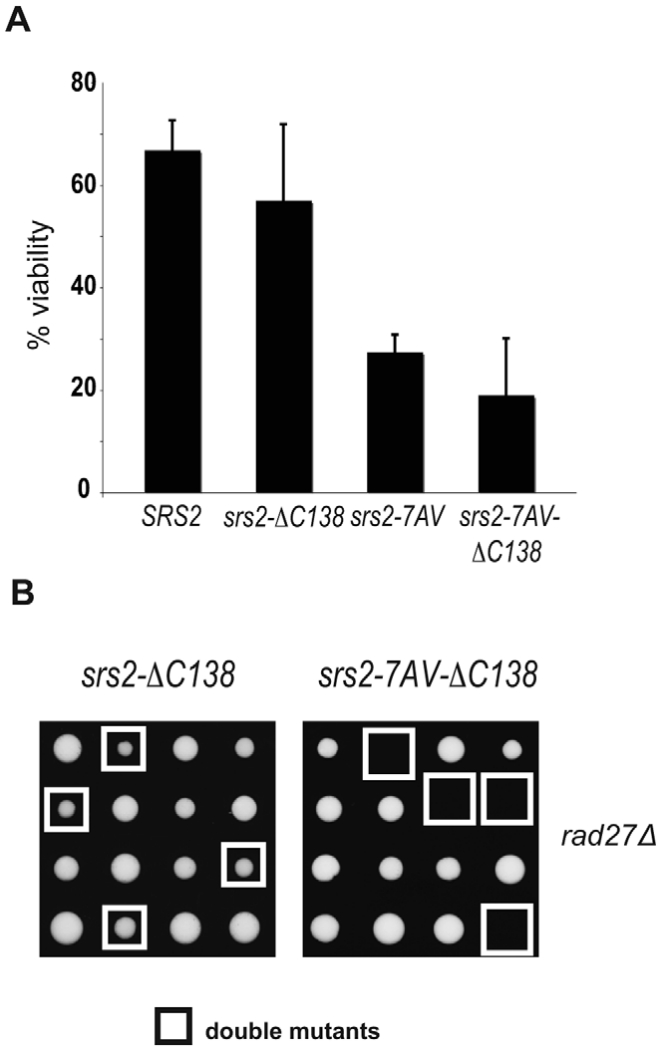
The Cdk1-dependent role of Srs2 does not depend on its interaction with PCNA. (A) Viability of *SRS2* and *srs2-7AV* strains, with and without the PCNA interacting domain, after DSB induction. (B) Tetrads obtained from sporulation of diploids heterozygous for *rad27*Δ and the indicated *srs2* mutations. Double mutant spores are indicated by the white squares.

The sumoylation consensus sites are located in the last 138 residues of the C-terminus tail of Srs2 (Figure 1A), which also mediates the interaction with PCNA [21]. Hence, we asked if this tail is important for the Cdk1-dependent role of Srs2. As shown in Figure 6, we found that the srs2*−ΔC138* mutant is viable after induction of a HO-mediated DSB and also when combined with a *rad27*Δ. Conversely, unphosphorylatable *srs2-7AV* mutants lacking the PCNA-interaction domain (*srs2-7AVΔC138*) are lethal in both contexts. These data suggest that Cdk1 targets Srs2 to promote recombinational repair independently of the interaction with PCNA and sumoylation. Moreover, elimination of sumoylation sites, but not deletion of the Srs2 tail containing the same sites, suppresses the recombination defects in the *srs2-7AV* mutant.

## Discussion

Increasing evidence suggests that cell cycle-dependent kinase Cdk1 is required for the DNA damage response [32], so changing the general view that in the presence of DNA lesions Cdk1 has to be inhibited to allow sufficient time for DNA repair. It rather suggests that Cdk1 is actively involved in DNA repair. Cdk1 phosphorylates Sae2, which is part of the DSB resection machinery, and is needed to promote HR [35]. Sae2 is the only Cdk1 target that functions in DSB repair identified thus far, although Cdk1 is required at later steps during HR [33]. Cdk1 also phosphorylates the budding yeast Srs2 protein [30],[31] a member of the evolutionarily conserved family of UvrD proteins, which displays both DNA unwinding [22] and sDNA translocation activities *in vitro* [18],[19]. The DNA translocase activity of Srs2 is essential to disrupt toxic Rad51 nucleofilaments and prevent unwanted recombination events, while the helicase activity of Srs2, both in yeast and plants, is thought to be required to dismantle DNA structures mimicking a D-loop [23],[24].

One key finding in the present study is that Cdk1-dependent phosphorylation of Srs2 is required to complete the SDSA pathway, thus Srs2 is a novel target for Cdk1 in DSB repair. Srs2 phosphorylation controls the quality of DSB repair by preventing crossover outcome. Since we found that Srs2 phosphorylation is not required for the removal of toxic Rad51 filaments, we suggest that Cdk1 targets Srs2 helicase to dismantle D-loop structures in order to favor non-crossover products.

### Srs2 phosphorylation promotes recombinational repair, but is dispensable for reversal of toxic Rad51 nucleofilaments at gaps

Recombination can be both prevented and stimulated in *srs2* mutants, suggesting a dual role for Srs2 in HR. The finding that Srs2 is a DNA translocase that antagonizes the formation of unscheduled Rad51 filaments explains certain *srs2* phenotypes in HR that are suppressed by ablating *RAD51*; these include the synthetic lethality with *sgs1* mutants or high sensitivity to UV-light [13],[16]. Nevertheless, *srs2* mutants are defective in Rad51-dependent DSB repair [25],[28] or lethal when combined with *rad27*Δ mutants [14],[41]. These are contexts in which HR is essential to restore DNA lesions and the activity of Srs2 is required to promote homologous recombinational repair.

In this study we analyzed the recombination phenotypes of two *srs2* mutants that mimicked either the constitutive unphosphorylated (*srs2-7AV*) or Cdk1-dependent phosphorylated (*srs2-7DE*) protein isoforms. We found that *srs2-7AV* unphosphorylatable mutants display only a subset of *srs2Δ* phenotypes and, in particular, they do not display those phenotypes that are suppressed by *RAD51* deletion. In fact, *srs2-7AV* mutants are not UV-sensitive or synthetically lethal with *sgs1*Δ, but are non-viable when combined with *rad27* mutants or treated with the DSB-inducing drug zeocin. Thus, functions of Srs2 in preventing unscheduled recombination or in allowing efficient recombinational repair are genetically separable. The phosphorylation of Srs2 is dispensable for the removal of toxic Rad51 nucleofilaments assembled at gaps, while it is essential to promote recombinational repair.

### Srs2 phosphorylation is required during DSB repair to complete the SDSA pathway

**Figure 7:**
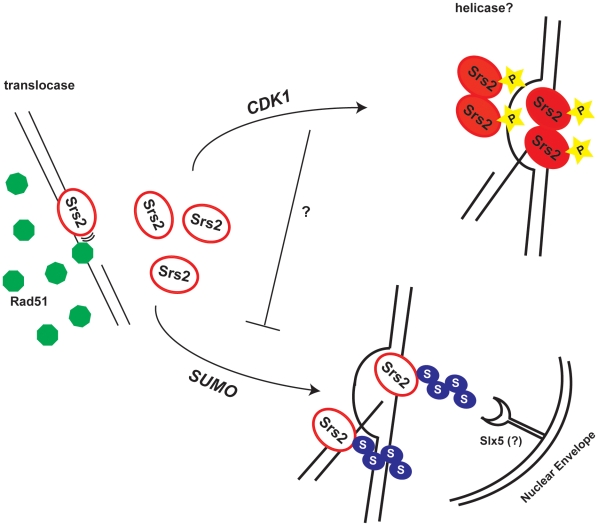
Model for the roles of the Srs2 modifications in recombination. Srs2 removes toxic Rad51 filaments by translocating on ss-DNA. In response to a DSB, Cdk1 targets Srs2 helicase to dismantle the D-loops, thus allowing SDSA pathway that limits DNA exchanges. When Srs2 phosphorylation is prevented, unscheduled sumoylation takes over and the DSB is channeled into the Slx5-dependent pathway.

In accordance with the finding that Srs2 phosphorylation is essential to promote recombination, we found that it is also required for Rad51-mediated DSB repair. In particular, we have been able to show that Srs2 phosphorylation is necessary to complete SDSA in DSB repair. ChIP data on Rad51 are consistent with the idea that strand invasion is not affected and that Rad51 protein does not persist on the D-loops in *srs2Δ* or *srs2-7AV* mutants, although we cannot rule out that presynaptic filament assembly may somehow be affected in the absence of Srs2 or its phosphorylation. ChIP analysis conducted on Srs2 suggests that the protein is found at DSBs upon strand invasion, thus it is likely loaded at D-loops. Taken together, these data are consistent with a role of phosphorylated Srs2 in SDSA pathway, but another helicase/translocase may be implicated in removing Rad51 at the D-loops. We favour the idea that Cdk1 targets Srs2 to dismantle the D-loop intermediate in SDSA (Figure 7) perhaps after DNA synthesis has extended the invading strand. Srs2 helicase activity might be stimulated by binding to the D-loop structure and/or by interaction with other recombination factors. ChIP data conducted on unphosphorylatable Srs2-7AV at the invading strand suggest that the mutated protein accumulates at later times and is not rapidly dislodged from DNA as the wild-type protein. The fact that unphosphorylatable Srs2 appears glued at the D-loops is evocative of a protein working very inefficiently and whose turnover is largely prevented. It is likely that the unscheduled accumulation of the protein on the DNA might contribute to impaired cell viability and, consistent with this idea, the lethal phenotype of *srs2-7AV* mutant in response to DSBs is dominant (Figure S2).

Our data indicate that the proportion of *srs2-7AV* cells that do not survive DSB repair via GC is higher than the one, which fails to repair DNA lesion (Figure 3). This suggests that a fraction of *srs2-7AV* cells might die because of checkpoint-mediated arrest, as in *srs2Δ* mutants [25]. However, Srs2 phosphorylation is not required for recovery during DSB repair by SSA, that is, when Srs2 is probably engaged to remove toxic Rad51-depedent DNA structures, rather than working at the D-loop intermediate [45]. Thus, the checkpoint recovery defect in *srs2-7AV* mutants might have different causes during DSB repair by GC or SSA; as described below, perhaps some aspects of recovery defect in *srs2* mutants in GC could be explained considering that Cdk1-dependent phosphorylation was no longer required for Srs2 recombination activity, if sumoylation is also prevented.

### Cdk1 activity prevents unscheduled sumoylation of Srs2

We found that Srs2 sumoylation can be detected *in vivo* in response to heavy DNA damage. Protein modification is prevented ablating three lysine residues located in the extreme C-terminus tail of Srs2. Sumoylation and Cdk1-dependent phosphorylation modifications of Srs2 are independent events, but when phosphorylation fails, sumoylated Srs2 accumulates. There is a functional relationship between these two DNA damage induced modifications, since ablation of sumoylation residues largely rescues the recombinational repair phenotypes of *srs2-7AV* mutants.

What may be the mechanism for the toxicity of sumoylation in the absence of phosphorylation? Sumoylation of Srs2 alone appears unnecessary for many of its recombination functions (D. Callahan and H.Klein, unpublished results); here we show that it is not essential in DSB repair (see Figure 5B and Figure 6). While the biological significance of Srs2 sumoylation waits to be elucidated, we speculate that it might be important for degradation of Srs2 protein. Srs2 can interact physically with Slx5 [39], that in complex with Slx8, has been implicated in degradation of sumoylated proteins bound to irreparable DNA breaks at the nuclear periphery [8],[9]. Our data suggest that Cdk1-dependent phosphorylation of Srs2 counteracts its sumoylation, which takes over only in response to massive DNA damage. Thus, in a possible scenario, unphosphorylated and sumoylated Srs2 is trapped at DSB and becomes channeled via the Slx5/Slx8 pathway to the nuclear periphery (Figure 7).

Since this emergency nuclear periphery pathway intervenes to degrade proteins in response to irreparable DSBs, it might normally act on phosphorylated and sumoylated Srs2 and, therefore, Srs2-7AV cannot be eliminated. Conversely, after successful DSB repair, phosphorylated Srs2 could be recycled by other routes, and independent of sumoylation. Intriguingly, the unscheduled Srs2-dependent sequestration of DSBs to the periphery might explain the checkpoint recovery defects in *srs2-7AV* and perhaps also that of *srs2Δ*, if we imagine that another unregulated DNA helicase takes over in the absence of Srs2. Our studies did not show any obvious alterations in Srs2 protein levels in *srs2* phospho-mutants and/or SUMO-mutants (M.Saponaro and G.Liberi, unpublished results), but local protein degradation events at damaged DNA could be relevant.

Elimination of sumoylation compensates for the absence of phosphorylation of Srs2 in DSB repair, but paradoxically this rescue requires the last 138 residues of Srs2 that are not normally necessary for DSB repair. Hence, this suppression might require interaction with other factors. Preventing sumoylation in the unphosphorylatable Srs2 rescues recombination defects that ensue after a single DSB, but the importance of these Srs2 modifications become evident when many breaks occur, as in the *rad27*Δ mutants.

### Roles of Srs2 modifications in replication-induced DNA damage

We found that Srs2 phosphorylation is essential for recombinational repair of spontaneous damage occurring during S-phase in *rad27*Δ mutants. Similar to the response to DSBs, sumoylation of Srs2 is a main cause of death in *srs2-7AV* phospho-mutants. It is more difficult to predict the kind of damage which requires phosphorylated Srs2 in *rad27*Δ mutants. Rad27 is required for Okazaki DNA fragment processing [48] and in its absence, Srs2 might dismantle DNA and/or RNA structures that block HR. In any case, based on our conclusion that Srs2 phosphorylation is not essential for the processing of toxic Rad51 filaments, we think it more probable that the helicase activity, rather than translocase activity, is crucial for the survival in *rad27*Δ mutants. This proposed role of phosphorylated Srs2 in replication might seem at odds with the role suggested for Srs2 in preventing recombinational repair during S-phase through recruitment by sumoylated PCNA [20],[21]. However, in PRR mutants, Srs2 is proposed to be recruited by PCNA to disrupt Rad51 filaments at DNA gaps, while in the absence of Rad27, we are considering that Srs2 acts in a PCNA-independent and phosphorylation-dependent role as a helicase, rather than as a translocase. Importantly, *srs2-7DE* mutants slightly suppress the MMS sensitivity of PRR mutants, suggesting that the phosphorylated Srs2 is less efficient as a DNA translocase than the non-phosphorylated isoform. This is unmasked in PRR mutants, where it is likely that many sDNA breaks occur. Srs2 phosphorylation might modulate its interaction with PCNA, a hypothesis that will be interesting to test in the future.

### Concluding remarks

Our data indicate that Srs2 is a new target of Cdk1 kinase in DSB repair, acting at the level of strand invasion, rather than during DNA end resection. Srs2 phosphorylation is required to limit the extent of DNA exchanges during DSB repair with a function that is genetically separable from its role in processing toxic Rad51 filaments. We suggest that Cdk1-mediated phosphorylation might control, throughout the interaction with PCNA and/or other factors, the ability of Srs2 to function as a translocase or a helicase that inhibits or allows HR depending on the context. Furthermore, our data unravel a novel aspect of Cdk1-dependent regulation in counteracting untimely sumoylation events, which might become toxic for recombination if not properly scheduled.

## Materials and Methods

### Strains and plasmids

Genotypes of the strains used in this study are listed in Table S1. Deletion strains were obtained by the one-step PCR method and multiple mutant strains were derived from meiotic segregants of appropriate crosses. The *srs2-7DE* phospho-mutant was constructed by a site-directed mutagenesis strategy already described to construct the *srs2-7AV* mutant [39]. Mutations in *SRS2* were introduced at the seven consensus sites for the Cdk1 kinase (T604D, S698E, S879E, S893E, S938E, S950E and S965E). Construction of *srs2-3KR* strain, containing mutations K1081R, K1089R and K1142R at SUMO-consensus sites, will be described in detail elsewhere. *srs2-7AV3KR* mutant was constructed as follow: a NAT selection cassette was integrated downstream of the *srs2-3KR* mutated gene. DNA primers were designed to amplify a DNA region containing both the 3KR mutations and the NAT cassette. This DNA region was then used to replace, by transformation, the C-terminus of the *srs2-7AV*. A similar PCR-mediated strategy was used to delete the C-terminus-PCNA interaction domain in both wild type and *srs2-7AV* mutants. *SRS2* and *srs2* mutants were also cloned into the low copy-number Ycplac22 vector by gap-repair procedure or using PCR-based strategies described above and were used in all HO-based experiments. As tested by Western blotting, protein levels are similar when Srs2 or its mutated versions were expressed from *SRS2* chromosomal locus or from the Ycplac22 centromeric plasmid.

### UV and drug treatments

Log-phase cells were spread on YPD plates, irradiated with UV light (254 nm) and incubated in the dark; cell survival was compared to that of untreated controls. Log-phase cultures were incubated with different doses of zeocin (Invitrogen) for 1 hour and cell survival was calculated by comparing the plating efficiency with untreated cells. The UV and zeocin curves are the average of three independent experiments. Spot assays were performed by evaluating the growth of serially diluted cultures on synthetic complete medium containing adenine at a final concentration of 0.7 g/liter, with or without MMS (SIGMA).

### DSB repair efficiency, crossover frequency, and viability measurements

Relative frequencies of survival of cultures plated on glucose and galactose using the SSA and GC DSB HO-inducible systems were calculated as previously described [25],[29]. Product formation and analysis of crossover formation were assessed by Southern blotting analyses as described in [25]. The results shown are the average of three to five independent experiments.

### ChIP experiments and quantitative PCR

Asynchronous cultures were grown overnight at 28°C in YEP media containing 2% raffinose. When cultures reached mid log-phase, 15 µg/ml nocodazole was added to synchronize the cells in G2/M. Expression of the *HO* endonuclease was induced by addition of 2% galactose for the indicated times. ChIP analysis was carried out as previously described [49]. Samples were incubated with 1% Formaldehyde for 20 min with the anti-Rad51 ChIP and 45 minutes with the anti-Srs2 ChIP. Immunoprecipitation was carried out with clarified extracts using anti-Rad51 (kindly gift of Patrick Sung, Yale University, New Heaven) or anti-Srs2 (Santa Cruz Biotechnology) antibodies overnight at 4°C. Levels of immunoprecipitated DNAs were measured by quantitative real-time PCR using the SYBR Green technique (SYBR Green PCR Master Mix, Applied Biosystems) and run in an Applied Biosystems 7500 Fast Real-Time PCR System. Sequences of the DNA primers are listed in the Table S2. Dissociation stage curves were checked to test primer specificity. The results were analyzed with the 2^-DC^_T_ method as previously described [50]. For the ChIP anti-Rad51 experiments the relative enrichment was determined by the fold increase of ChIPed DNA relative to that prior to DSB induction; for the ChIP anti-Srs2 the absolute 2^-DC^_T_ variation after the subtraction of the 2^-DC^_T_ of the ChIP carried out in parallel in a *srs2Δ* mutant is shown. The total amount of DNA is normalized respect to an unrelated locus near *ARS305*.

### Protein analysis and detection of the sumoylated forms

Proteins were extracted using a TCA protocol [30]. Western blotting analysis was performed as previously described [30] with anti-Rad53 [51] and anti-Srs2 (Santa Cruz Biotechnology) antibodies. Immuno-precipitation of Srs2 was carried out with an anti-Srs2 antibody or without antibody as a control on proteins extracted by the TCA protocol using a 50 ml culture of cells at a density of 10^7^ cells/ml. After a series of washings with JS buffer (50 mM Hepes pH 7,5, 150 mM NaCl, 1,5 mM MgCl2, 1% glycerol, 5 mM EGTA, 1% Triton X-100), proteins were resuspended in a suitable volume of Laemmli buffer and separated on a 10% acrylamide SDS-PAGE gel. The blots were probed with a rabbit anti-SUMO antibody (kindly gift of Xiaolan Zhao, Memorial Sloan-Kettering Cancer Center, New York) and a HRP-labeled secondary antibody (Amersham). Blots were then stripped with the commercial solution Restore Western Blot Stripping Buffer (Thermo Scientific) and probed with the anti-Srs2 antibody (Santa Cruz Biotechnology).

## Supporting Information

Figure S1 Analysis of Srs2 sumoylation and its interplay with phosphorylation. (A) Analysis of Srs2 sumoylation was performed in E3 ligase deficient mutants, as described in Figure 5. (B) The DNA damage-induced Srs2 phosphorylation was evaluated in *SRS2* and *srs2-3KR* mutants upon exposure to 0.02% MMS for 3 hours.
(0.22 MB TIF)

Figure S2 The lethal phenotype in recombinational repair of *srs2-7AV* mutants is dominant. Cell survival in response to DSB induction were evaluated in the presence of a genomic copy of *SRS2* and *srs2* phospho-mutants carried on a low copy number plasmid.
(0.05 MB TIF)

Table S1 Yeast strains used in this study.
(0.09 MB DOC)

Table S2 DNA primers used in ChIP and Q-PCR experiments.
(0.03 MB DOC)
