# Supplementary figures and images for: Cdk1 Targets Srs2 to Complete Synthesis-Dependent Strand Annealing and to Promote Recombinational Repair
Source: PLoS Genet. 2010 Feb 26;6(2):e1000858. doi: 10.1371/journal.pgen.1000858 (PMC2829061; doi:10.1371/journal.pgen.1000858)

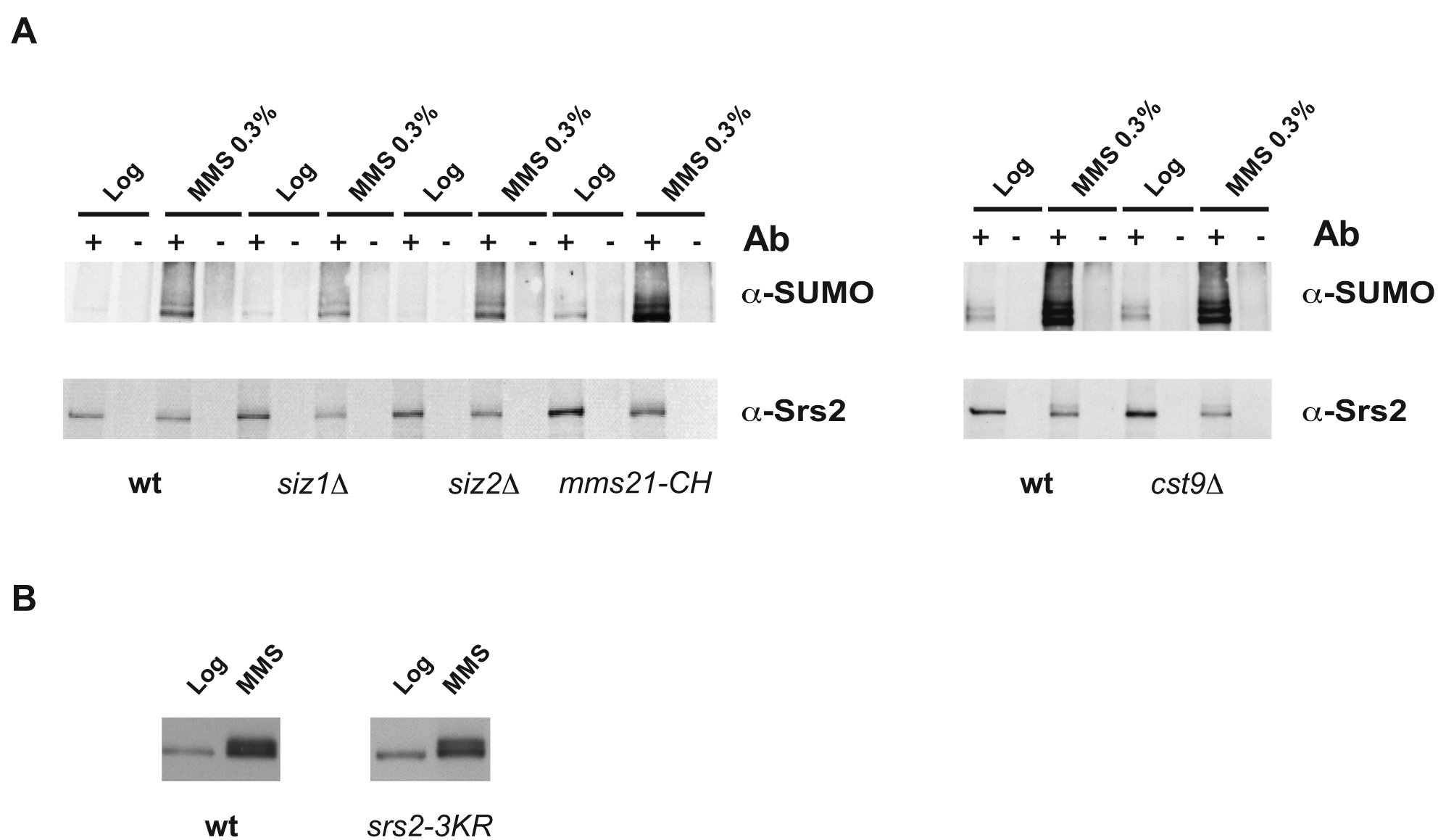

Supplement: Figure S1 — Analysis of Srs2 sumoylation and its interplay with phosphorylation. (A) Analysis of Srs2 sumoylation was performed in E3 ligase deficient mutants, as described in Figure 5. (B) The DNA damage-induced Srs2 phosphorylation was evaluated in SRS2 and srs2-3KR mutants upon exposure to 0.02% MMS for 3 hours. (0.22 MB TIF) [file pgen.1000858.s001.tif]

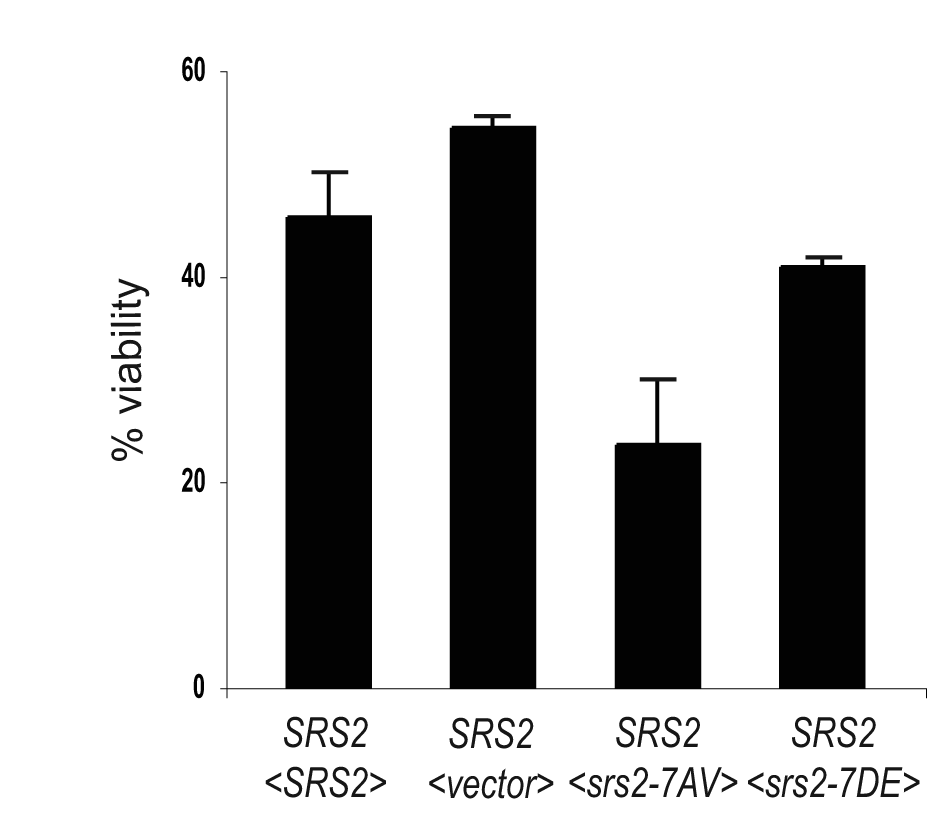

Supplement: Figure S2 — The lethal phenotype in recombinational repair of srs2-7AV mutants is dominant. Cell survival in response to DSB induction were evaluated in the presence of a genomic copy of SRS2 and srs2 phospho-mutants carried on a low copy number plasmid. (0.05 MB TIF) [file pgen.1000858.s002.tif]
