# Supplementary material for: Cdk1 Targets Srs2 to Complete Synthesis-Dependent Strand Annealing and to Promote Recombinational Repair
Source: PLoS Genet. 2010 Feb 26;6(2):e1000858. doi: 10.1371/journal.pgen.1000858 (PMC2829061; doi:10.1371/journal.pgen.1000858)
Supplement: Table S1 — Yeast strains used in this study. (0.09 MB DOC) [file pgen.1000858.s003.doc]

Supplementary Table S1. *Saccharomyces cerevisiae* strains used in this study.

| **Strain** | Relevant genotype | Source |
| --- | --- | --- |
| **SY2080** | *MATa ade2-1 trp1-1 leu2-3112 his3-1115 ura3 can1-100 GAL PSI1^+^Rad5+* | H. Klein |
| ***srs2-3KR*** | *W303 MATa srs2-3KR* | H. Klein |
| **CY9473** | *W303 MATa srs2-3KR-NAT* | This study |
| **SY2215** | *W303 MATalpha rad5::URA3* | H. Klein |
| **CY9013** | *W303 MATalpha rad27::TRP1* | This study |
| **YMV80** | *matΔ::hisG1, hmlΔ::ADE, hmrΔ::ADE1, lys5,ura3-52, leu2::HOcs, ade3::GAL::HO, his-URA3-5’Dleu2-is4* | (Vaze et al., 2002) |
| **tGI354** | *Δho Δhml::ADE1, MATainc, Δhmr::ADE1, ade1, leu 2-3,112, lys 5, trp::his G, ura 3-55, ade3::GAL::HO, arg 5,6::MATa:HYG* | (Ira et al., 2003) |
| **mms21-CH** | *MATa* *ade2-1 can1-100 his3-11,-15 leu2-3,112 trp1-1 ura3-1 mms21-C200A/H202A-13Myc::HIS3* | D. Branzei |
| **Wt**  **BY4741** | *MAT alpha can1Δ::STE2pr-Sp_his5 lyp1Δhis3Δ1 leu2Δ0 ura3Δ0 met15Δ0 LYS2+* | Lab stock |
| **cst9Δ** | *MAT alpha can1Δ::STE2pr-Sp_his5 lyp1Δhis3Δ1 leu2Δ0 ura3Δ0 met15Δ0 LYS2+ cst9:: KanMX4* | Lab stock |
| **CY8626** | *W303 MATalpha sgs1::KanMX4* | Lab stock |
| **CY2643** | *W303 MATa srs2::KanMX4* | Lab stock |
| **CY5616** | *YMV80 MATa srs2::LEU2 <TRP1, CEN>* | This study |
| **CY5617** | *YMV80 MATa srs2::LEU2 <SRS2, TRP1, CEN>* | This study |
| **CY5621** | *YMV80 MATa srs2::LEU2 <srs2-7AV, TRP1, CEN>* | This study |
| **CY5623** | *YMV80 MATa srs2::LEU2 <srs2-7DE, TRP1, CEN>* | This study |
| **CY6136** | *W303 MATa srs2::srs2-7AV-HIS3MX6* | Chiolo et al. 2005 |
| **CY6139** | *W303 MATa srs2::srs2-7DE-HIS3MX6* | This study |
| **CY9630** | *W303 MATa srs2::srs2-7AV3KR-NAT* | This study |
| **CY7969** | *W303 MATa srs2::HIS3MX6, rad5::URA3* | This Study |
| **CY8336** | *W303 MATa siz1::KanMX4* | Lab Stock |
| **CY8337** | *W303 MATa siz2::KanMX4* | Lab Stock |
| **CY8568** | *W303 MATa srs2::srs2-7DE-HIS3MX6 rad5::URA* | This Study |
| **CY8573** | *W303 MATa srs2::srs2-7AV-HIS3MX6 rad5::URA* | This Study |
| **CY9459** | *tGI354 MATa srs2::LEU2 <SRS2, TRP1, CEN>* | This Study |
| **CY9461** | *tGI354 MATa srs2::LEU2 <TRP1, CEN>* | This Study |
| **CY9463** | *tGI354 MATa srs2::LEU2 <srs2-7AV, TRP1, CEN>* | This Study |
| **CY9465** | *tGI354 MATa srs2::LEU2 <srs2-7DE, TRP1, CEN>* | This Study |
| **CY9468** | *tGI354 MATa srs2::LEU2 <srs2-7AV-3KR-NAT, TRP1, CEN>* | This Study |
| **CY9469** | *tGI354 MATa srs2::LEU2 <srs2-3KR-NAT, TRP1, CEN>* | This Study |
| **CY9602** | *tGI354 MATa srs2::LEU2 <srs2-ΔC138-KanMX4, TRP1, CEN>* | This Study |
| **CY9604** | *tGI354 MATa srs2::LEU2 <srs2-7AV-ΔC138-KanMX4, TRP1, CEN>* | This Study |
| **CY9471** | *W303 MATa srs2::srs2-ΔC138::KanMX4* | This Study |
| **CY9612** | *W303 MATa srs2-7AV-ΔC138::KanMX4* | This Study |
| **CY8563** | *W303 MATalpha srs2::KanMX4 rad18::LEU2* | This Study |
| **CY8564** | *W303 MATalpha rad18::LEU2* | This Study |
| **CY8566** | *W303 MATa srs2:: srs2-7AV-HIS3MX6 rad18::LEU2* | This Study |
| **CY8570** | *W303 MATa srs2::srs2-7DE-HIS3MX6 rad18::LEU2* | This Study |
