# Supplementary material for: Cdk1 Targets Srs2 to Complete Synthesis-Dependent Strand Annealing and to Promote Recombinational Repair
Source: PLoS Genet. 2010 Feb 26;6(2):e1000858. doi: 10.1371/journal.pgen.1000858 (PMC2829061; doi:10.1371/journal.pgen.1000858)
Supplement: Table S2 — DNA primers used in ChIP and Q-PCR experiments. (0.03 MB DOC) [file pgen.1000858.s004.doc]

Supplementary Table S2. Primer sequence used for the Real-Time PCR.

| **Primer name** | **DNA sequence** |
| --- | --- |
| ARS501-1 | tgcaaacagtattccggcac |
| ARS501-2 | ACACGATCCACGCTGTCCCA |
| + 0,2 kb F | cctggttttggttttgtagagtgg |
| + 0,2 kb R | GAGCAAGACGATGGGGAGTTTC |
